# Lignin peroxidase functionalities and prospective applications

**DOI:** 10.1002/mbo3.394

**Published:** 2016-09-07

**Authors:** Ayodeji O. Falade, Uchechukwu U. Nwodo, Benson C. Iweriebor, Ezekiel Green, Leonard V. Mabinya, Anthony I. Okoh

**Affiliations:** ^1^ SAMRC Microbial Water Quality Monitoring CentreUniversity of Fort HareAliceSouth Africa; ^2^ Applied and Environmental Microbiology Research Group (AEMREG)Department of Biochemistry and MicrobiologyUniversity of Fort HareAliceSouth Africa

**Keywords:** decolourization, lignin peroxidase, ligninolytic enzymes, melanin oxidation, peroxidases

## Abstract

Ligninolytic extracellular enzymes, including lignin peroxidase, are topical owing to their high redox potential and prospective industrial applications. The prospective applications of lignin peroxidase span through sectors such as biorefinery, textile, energy, bioremediation, cosmetology, and dermatology industries. The litany of potentials attributed to lignin peroxidase is occasioned by its versatility in the degradation of xenobiotics and compounds with both phenolic and non‐phenolic constituents. Over the years, ligninolytic enzymes have been studied however; research on lignin peroxidase seems to have been lagging when compared to other ligninolytic enzymes which are extracellular in nature including laccase and manganese peroxidase. This assertion becomes more pronounced when the application of lignin peroxidase is put into perspective. Consequently, a succinct documentation of the contemporary functionalities of lignin peroxidase and, some prospective applications of futuristic relevance has been advanced in this review. Some articulated applications include delignification of feedstock for ethanol production, textile effluent treatment and dye decolourization, coal depolymerization, treatment of hyperpigmentation, and skin‐lightening through melanin oxidation. Prospective application of lignin peroxidase in skin‐lightening functions through novel mechanisms, hence, it holds high value for the cosmetics sector where it may serve as suitable alternative to hydroquinone; a potent skin‐lightening agent whose safety has generated lots of controversy and concern.

## 1 Introduction

The espousal for the utilization and perhaps, the utilization of lignocellulosic biomass for the production of value‐added products is on the increase world over, partly, due to the abundance and renewable nature of lignocellulosic biomass. Woody and nonwoody plants possess lignocellulose as major structural components and two carbohydrate polymers viz. cellulose (~30–50%) and hemicelluloses (~15–30%) as well as some non‐carbohydrate aromatic polymers (~15–30%) constitute lignocellulose (Foyle, Jennings, & Mulcahy, 2007; Harris & Debolt, 2010; Menon & Rao, 2012). In woody or herbaceous plants, the lignocellulose constituent varies in accordance with the species and in tandem with the biotic and abiotic stressor factors including environmental distress syndromes.

Several categorizations has applied to lignocellulosic biomass including waste biomass, virgin biomass, and energy crops. Waste biomass are thought to be low‐value by‐products largely generated from industrialized forestry activities (sawdust, wood waste, pulp mill waste), agricultural practices (corn stover/cob, sugarcane bagasse, wheat straw, rice husks, animal droppings), and municipal solid waste. On the other hand, terrestrial plants are classified as virgin biomass while energy crops include those generating large amount of lignocellulosic biomass as feedstock for second‐generation biofuel production.

Increased generation of lignocellulosic wastes from both the industrial and agricultural sectors have continued to pose environmental challenge globally due to, in part, poor waste management. However, the prospect of the valorization of lignocellulosic wastes for value‐added products shall suffice as effective waste management strategies. Nonetheless, this proposition is at the moment underexplored.

Besides, valorization of lignocellulosic wastes avails the right set of circumstance for the harnessing of value‐added products from the compositional structures of the lignocellulosic biomass. The valorized may be, in effect, the products of interest for end users, otherwise, they may serve as raw materials for the production of commercially viable products. On the converse, microbial activities on lignocellulosic biomass during valorization process may also generate industrially important products including enzymes, ethanol, organic acids, microbial polysaccharides, and vitamins. The interest in enzymes is informed by their enormous industrial and biotechnological applications. However, the cost of enzyme production has continued to increase; thus, the need for a cost‐effective means of production is imperative. Nonetheless, an important cost reduction strategy for enzyme production includes the exploration of alternative cheap carbon sources for fermentation, and lignocellulosic biomass may serve the purpose of cheap carbon source. Abundance, availability, and renewable nature bestow lignocellulosic biomass the status of near‐perfect candidature of a cheap carbon source. Consequently, a variety of lignocellulosic materials have been utilized for different enzyme production processes (Asgher, Iqbal, & Asad, 2012; Asgher, Iqbal, & Irshad, 2012; Kang, Park, Lee, Hong, & Kim, 2004; Knezevic, Milovanovic, Stajic, & Vakojevic, 2013; Reddy, Babu, Komaraiah, Roy, & Kothari, 2003), and a conspectus of some of these processes has been articulated in Table 1.

**Table 1:** Valorization of some lignocellulosic biomass for ligninolytic and cellulolytic enzyme production

Lignocellulosic Biomass	Microorganism	Enzymes Produced	References
Rice straw	*P. chrysosporium*; *T. versicolor*; *Trichoderma reesei*; *Aspergillus niger* KK2	LiP, MnP, Laccase, Cellulases, Hemicellulases	Kang et al. (2004), Iqbal, Ahmed, Zia, and Irfan (2011), Asgher, Ahmad, and Iqbal (2011), Saratale et al. (2014)
Sugarcane bagasse	*Thermoascus aurantiacus*; *Bacillus circulans*; *Trametes villosa*	Xylanase, MnP	Milagres, Santos, Piovan, and Roberto (2004), Bocchini, Oliveira, Gomes, and Da Silva (2005), Silva et al. (2014)
Wheat straw	*Phlebia radiata*; *Trichoderma viride*; *Trametes suaveolens*	LiP, MnP, Laccase, Cellulase	Vares, Kalsi, and Hatakka (1995), Iqbal et al. (2011), Knezevic et al. (2013)
Banana waste	*P. ostreatus*; *P. sajor‐caju*; *Schizophyllum commune* IBL‐06	LiP, MnP, Laccase, Xylanase, Endoglucanase, Exoglucanase	Reddy et al. (2003), Irshad and Asgher (2011), Asgher, Irshad, and Iqbal (2012)
Corn cobs	*Trametes versicolor*	LiP, MnP, Laccase	Asgher, Iqbal, & Asad (2012); Asgher, Iqbal, & Irshad (2012)
Sawdust	*Trametes suaveolens*	MnP, Laccase	Knezevic et al. (2013)
Pea pods	*Aspergillus niger* HN‐1	Filter Paper Cellulase (FPase)β‐ glucosidase (BGL)	Sharma, Rawat, Bhogal, and Oberoi (2015)

LiP, Lignin Peroxidase; MnP, Manganese Peroxidase.

Irrespective of any delineated path that may lead to products of interest, lignin recalcitrance to degradation has remained a major bottleneck to various industrial operations. Besides the conferral of hydrolytic stability and structural rigidity to plant's cell walls, lignin traps and renders unavailable the saccharides constituting the mono‐, di‐, oligo‐ and poly‐meric units of cellulose necessary for fermentation. Lignin is imperative for the survival of plants and its recalcitrance to degradation has been attributed to its cross linkages with polysaccharides (cellulose and hemicellulose) via ester and ether linkages and as well as, its molecular architecture, in which various non‐phenolic phenylpropanoid uints produce a complicated three‐dimensional network joined by an array of ether and carbon–carbon bonds (Ruiz‐Dueñas & Martinez, 2009).

In a bid to address the challenge of lignin recalcitrance to degradation, several physicochemical pretreatment technologies have been developed to disrupt the non‐cellulosic matrix and render cellulose and hemicellulose more accessible for enzymatic hydrolysis (Mosier et al., 2005). Some of these methods include steam explosion, ammonia fiber explosion, acid hydrolysis, alkaline hydrolysis, ozonolysis, organosolvation, and oxidative delignification (Chaturvedi & Verma, 2013). These pretreatment technologies are generally expensive, require high energy inputs, generate compounds inhibitory to fermentation, releases toxic chemicals which leads to corrosion problems and may also lead to material loss (Chaturvedi & Verma, 2013; Huang, Santhanam, Badri, & Hunte, 2013). Nonetheless, biological method of delignification may serve as an alternative pretreatment process as it is saddled with fewer limitations (Huang et al., 2013; Kuhar, Nair, & Kuhad, 2008). Biological pretreatment involves the use of microorganisms or immobilized microbial submolecules such as enzymes. The method may be thought of as cheap and environmental‐friendly. However, it is not without demerits which include utilization of, part of, the fermentable sugars as carbon source consequently, lowering product yield (Potumarthi, Baadhe, Nayak, & Jetty, 2013; Wan & Li, 2011).

Lignin degradation has been extensively studied in wood‐rotting organisms, especially white‐rot basidiomycetes (Hatakka, 1994; Leonowicz et al., 1999; Martinez et al., 2004; Wan & Li, 2012), and most of these studies established white‐rot fungi as the most effective “delignifyer” partly as a result of the potent ligninolytic extracellular oxidative enzymes (ligninases) produced (Glenn, Morgan, Mayfield, Kuwahara, & Gold, 1983; Tien & Kirk, 1983). The ligninolytic extracellular oxidative enzymes have been classified into phenol oxidases and heme peroxidases. Enzymes in the phenol oxidases include laccases (EC 1.10.3.2) while the heme peroxidases include lignin peroxidase (EC 1.11.1.14), manganese peroxidase (EC 1.11.1.13), versatile peroxidase (EC 1.11.1.16), and dyP‐type peroxidases (EC 1.11.1.19). Also implicated in the degradation of lignin are some accessory enzymes such as aryl‐alcohol oxidase (EC 1.1.3.7), glyoxal oxidase (EC 1.2.3.5), and glucose 1‐oxidase (EC 1.1.3.4) which generate the hydrogen peroxide (H_2_O_2_) required by the peroxidases (Ander & Marzullo, 1997; Guillen, Martinez, & Martinez, 1992; Kersten & Kirk, 1987).

Lignin‐modifying enzymes (LMEs) have also shown capability toward the degradation of various xenobiotics including dyes, chlorophenols, polycyclic aromatic hydrocarbons (PAHs), organophosphorous compounds, and phenols (Wesenberg, Kyriakides, & Agathos, 2003). The high redox potentials of ligninases and their ability to oxidize materials recalcitrant to degradation motivates for their prospects in biopulping and biobleaching (Call & Call, 2005), bioremediation through textile dye transformation (Husain, 2010; Mehta, 2012), decolourization of distillery effluent and other waste effluent treatment (Rajasundari & Murugesan, 2011) and as well, the degradation of herbicides (Pizzul, Castillo, & Stenström, 2009). Consequently, the interest in the application of ligninases for biotechnological purposes continues and the imperativeness of the industrial potential is an indication of the value of these enzymes. This review, therefore, succinctly presents the functionalities of lignin peroxidase and also prospects into futuristic applications.

## 2 Peroxidases (EC 1.11.1)

Peroxidases catalyze the oxidation of various organic and inorganic substrates in the presence of hydrogen peroxide as electron acceptor; typical a reaction is as shown below.

$$2S+H_{2}O_{2}+2e^{-}\to 2S_{\text{ox}}+2H_{2}O$$

S, substrate (electron donor), S_ox_, oxidized substrate.

Peroxidases are distributed widely in nature; vast presence in plants, animals, and microbes have been documented (Battistuzzi, Bellei, Bortolotti, & Sola, 2010). They are grouped as heme and non‐heme peroxidases. The heme peroxidases contain a protoporphyrin IX (heme) as prosthetic group while the non‐heme peroxidases lack such prosthetic group. A recent classification phylogenetically divides heme peroxidases into two superfamilies (peroxidase‐cyclooxygenase superfamily and peroxidase‐catalase superfamily) and three families including di‐heme peroxidases, dyP‐type peroxidases (DyPPrx), and haloperoxidases (HalPrx), respectively (Zamocky & Obinger, 2010).

The Peroxidase‐cyclooxygenase superfamily is made up of members from all domains of life (Zamocky et al., 2015) as against the old nomenclature “animal heme‐dependent peroxidases” which formerly restricted classification to only peroxidases of animal origin. They seem to dominantly catalyze halide oxidation (Zamocky et al., 2015). Several representatives of peroxidase‐cyclooxygenase superfamily are involved in the innate immune system (Söderhall, 1999). This function is not restricted to mammalian peroxidases alone as several peroxidases of bacterial origin (Dick et al., 2008) are suspected to be involved in unspecific defense mechanisms (Zamocky & Obinger, 2010). The involvement of the peroxidase‐cyclooxygenase superfamily in immunology would be of clinical significance. However, this is out of scope of this present review.

The peroxidase‐catalase superfamily may be further subdivided into three classes (Welinder, 1992). Class I involve intracellular peroxidases such as yeast cytochrome c peroxidase (CcP) which protects against toxic peroxide in the electron transport chain (Dunford, 1999), and recent evidences also suggest that this peroxidase functions as a mitochondrial peroxide sensing and signaling protein in *Saccharomyces cerevisiae* (Martins, Kathiresan, & English, 2013). Ascorbate peroxidase (APx) comes next, and it is associated with the removal of hydrogen peroxide in the chloroplast and cytosol of higher plants (Battistuzzi et al., 2010; Dunford, 1999) and lastly, the bacterial catalase‐peroxidase (KatG) which is known to exhibit hybrid catalytic activities of both peroxidase and catalase and, they are thought to have cell protective fender under oxidative stress (Battistuzzi et al., 2010; Smulevich, Jakopitsch, Droghetti, & Obinger, 2006; Welinder, 1991). Class II, of the peroxidase‐catalase superfamily, are extracellular fungal peroxidases including lignin peroxidase (LiP), manganese peroxidase (MnP), and versatile peroxidase (VP) which are involved in lignin degradation while class III includes peroxidases secreted by plants such as horseradish peroxidase (HRP) which have been implicated in cell wall biosynthesis, Indole‐3‐acetic acid catabolism and oxidation of poisonous compounds (Battistuzzi et al., 2010; Veitch & Smith, 2001). The pertinent features of class 11 peroxidases motivate for the extensive inroad into the activities of heme peroxidases as have been synopsized in this review.

## 3 Class II Heme‐Peroxidases

Class II heme‐peroxidases are reported as fungal or bacterial in nature. They are extracellular enzymes associated with lignin degradation and, perhaps, portend vital roles in the valorization of lignocellulosic biomass to commercializable products. Ligninolytic heme‐peroxidases including MnP, LiP, and VP play central role in delignification (Ruiz‐Dueñas & Martinez, 2009). These peroxidases; MnP, LiP, and VP, oxidize specific components of the lignin structure and may act in synergy if they are produced by same organism. While MnP oxidizes the phenolic structures of lignin and LiP targets the non‐phenolic components, VP has the capability of oxidizing both phenolic and non‐phenolic structures.

MnP (manganese‐dependent peroxidase) was discovered by Kuwahara, Glenn, Morgan, and Gold (1984) and has been described as the most common lignin‐modifying peroxidase secreted by most white‐rot fungi and litter decomposers (Hofrichter, 2002). Its involvement in lignin degradation has been reported and well‐studied in fungi (Hofrichter, 2002), however, paucity of information exists on MnP‐producing bacteria. The mechanism of action of MnP includes the catalytic oxidation of Mn^2+^ to Mn^3+^, which is highly reactive and in turn oxidizes a wide range of phenolic substrates including lignin phenolic structures (Tuor, Wariishi, Schoemaker, & Gold, 1992). Nonetheless, MnP also possesses the capability to oxidize or cleave non‐phenolic structures with the contributions of mediators including thiyl or lipid radicals (Abdel‐Hamid, Solbiati, & Cann, 2013; Reddy, Sridhar, & Gold, 2003). Moreso, the ability of MnP to oxidize and depolymerize natural and synthetic lignin and as well, recalcitrant compounds has been reported (Bogan, Lamar, & Hammel, 1996; Dehorter & Blondeau, 1993; Hofrichter, 2002; Hofrichter, Steffen, & Hatakka, 2001; Hofrichter, Ullrich, Pecyna, Liers, & Lundell, 2010). MnPs possess two or three residues corresponding to Glu‐35, Glu‐39 and Asp‐175 of *Phanerochaete chrysosporium* MnP 1 that binds Mn (Floudas et al., 2012; Ruiz‐Dueñas et al., 2009).

LiP possesses high redox potential for the oxidation of non‐phenolic structures which constitute up to 90% of lignin (Martinez et al., 2005). It is also characterized with the ability to oxidize a wide range of aromatic compounds, hence, its role in the enzymatic degradation of lignin. Besides the characteristic oxidation of non‐phenolic substrates, LiP has also shown the capability to oxidize a variety of phenolic compounds (Baciocchi et al., 2001). Furthermore, VP, also known as hybrid peroxidase or manganese‐lignin peroxidase is characterized with a versatile ligninolytic ability as it combines the catalytic properties of both MnP (oxidation of phenolic substrates by oxidizing Mn^2+^ to Mn^3+^) and LiP (oxidation of non‐phenolic aromatic compounds). The unique molecular architecture of VP, which is characterized by the presence of different oxidation‐active sites, perhaps has been adduced to its versatility in activity. The ability of VP to oxidize high redox potential compounds is linked to an exposed catalytic tryptophan which forms a radical on the surface of the enzyme through a long‐range electron transfer to the heme (Ruiz‐Dueñas et al., 2009). This has recently been shown in a study by Saez‐Jimenez et al. (2015) where direct electron transfer from lignin polymer to a surface tryptophanyl radical of VP was responsible for lignin oxidation. Hence, the significance of VP Trp‐164 in delignification is pivotal. The focus on LiPs, in this review, is motivated by their high redox potential for the oxidation of non‐phenolic substrates and their emerging application in the treatment of hyperpigmentation and skin lightening.

## 4 Lignin Peroxidase (EC 1.11.1.14)

**Figure 1:**
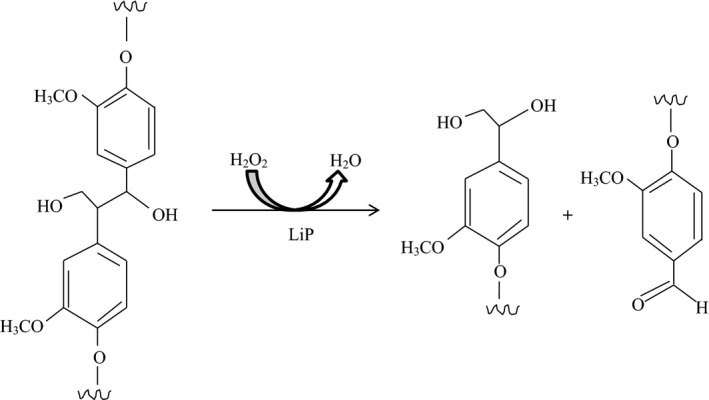
Oxidative cleavage of β‐1 linkage in lignin structure by lignin peroxidase (LiP)

Lignin peroxidase (LiP) is also referred to as diaryl propane oxygenase and it is a heme‐containing enzyme that catalyzes hydrogen peroxide‐dependent oxidative degradation of lignin (Fig. 1). Ligninase I similarly serve same function as diaryl propane peroxidase. These enzymes are inclusive of the peroxidase‐catalase superfamily (Zamocky & Obinger, 2010). Structurally, LiP is a monomeric hemoprotein. The nonplanarity of the heme cofactor of LiP and those in the other class‐II peroxidases has been well documented (Piontek, Glumoff, & Winterhatter, 1993), and observable in the structures of the different ligninolytic peroxidases deposited in the Protein Data Bank (PDB).

After the discovery of LiP in extracellular medium of white‐rot fungus; *P. chrysosporium* (Glenn et al., 1983; Tien & Kirk, 1983), various isozymes have been identified in the following organisms: *P. chrysosporium* (Farrell, Murtagh, Tien, Mozuch, & Kirk, 1989), *Tramates versicolor* (Johansson, Welinder, & Nyman, 1993), *Phlebia radiata* (Moilanen, Lundell, Vares, & Hatakka, 1996), and *Phanerochaete sordida* (Sugiura et al., 2009). Farrell et al. (1989) demonstrated the existence of six (6) isozymes of LiP designated H1, H2, H6, H7, H8, and H10 in the extracellular fluid of cultures of *P*. *chrysosporium* BKM‐F‐1767. Another isozyme of lignin peroxidase, Ha was later identified by Dass and Reddy (1990). In the same vein, Glumoff et al. (1990) characterized five (5) isozymes of LiP also from *P. chrysosporium*. The study reported that the purified isozymes had different isoelectric point, sugar content, substrate specificity, and stability. The N‐terminal sequences of their amino acids were also reported to be different which suggested that they were encoded by different genes. Gene sequencing of a lignocellulose degrading fungus; *P. chrysosporium* strain RP78, revealed about 10 *lip* genes, thus confirming the existence of isozymes of LiP (Martinez et al., 2004). Furthermore, Morgenstern, Klopman, and Hibbett (2008), in consistence with previous studies, reported *P. chrysosporium* genome to harbor 10 *lip* genes designated *lip* A‐J and they, respectively, encode different isoforms of lignin peroxidase. At the moment, the US Department of Energy Joint Genome Institute (http://www.jgi.doe.gov) is consistently updated with lignocellulolytic fungi genome sequences. As recent as 2015, new genes coding for ligninolytic peroxidases, including different LiP isoforms, have been identified (Couturier et al., 2015; Hori et al., 2014; Ruiz‐Dueñas et al., 2013).

Besides the white‐rot and brown‐rot fungi, some bacteria have also been reported to possess ligninolytic abilities with the potential of producing ligninases. This group of bacteria has been classified as actinomycetes, α‐proteobacteria, ___‐proteobacteria (Bugg, Ahmad, Hardiman, & Singh, 2011; Paliwal, Rawat, Rawat, & Rai, 2012). However, bacterial lignin peroxidase has been less studied, thus espousing the need to bioprospect for bacteria species with the ability to produce novel lignin peroxidase with broad spectrum and high enzyme activity.

## 5 Lignin Peroxidase Structure

LiP folds to form a globular shape with a size of about 50 × 40 × 40 Å (Piontek et al., 1993). It is segregated into proximal and distal domains by the heme which is completely fixed in the protein but made accessible through two small channels. The LiP folding motif contains eight major α‐helices, eight minor helices, and three short antiparallel β sheets (Choinowski, Blodig, Winterhalter, & Piontek, 1999). Overall, the catalytic cycle of LiP is comparable to that of typical heme‐peroxidases. However, structurally some differences between lignin peroxidase and other heme‐peroxidases exist. Similarly, the molecular weight range of lignin peroxidase has been documented as 38 kDa to 43 kDa, isoelectric point range of 3.3 to 4.7 (Glumoff et al., 1990; Kirk, Croan, Tien, Murtagh, & Farrell, 1986), and a very low optimum pH of around pH 3.0 with veratryl alcohol as the substrate (Furukawa, Bello, & Horsfall, 2014; Tien & Kirk, 1988). The low optimum pH of LiP distinguishes it from other peroxidases.

Crystallographic studies of cytochrome c peroxidase (CcP) and LiP revealed some structural differences; LiP possesses four disulfide bonds while CcP has none. LiP is larger in size and contains about 343 amino acid residues while CcP is made up of 294 residues (Edwards, Raag, Wariishi, Gold, & Poulos, 1993). However, CcP is thought to be abundantly endowed with oxidizable amino acids (seven tryptophans, 14 tyrosine residues, five methionines, and one cysteine) and in contrast, LiP has three tryptophans and eight methionines. Tyrosine is absent in LiP and it also does not have free cysteine. Nonetheless, a very notable difference between LiP and CcP includes the presence of phenylalanines at the contact point between the distal and proximal heme surfaces in LiP and the replacement of phenylalanines with tryptophans in the case of CcP. Similarly, Asp‐183 is hydrogen bonded to heme propionate in LiP while Asn‐184 plays this role in CcP. This has been suggested to partly account for the low pH optimum of lignin peroxidase as the disruption of the aspartic acid‐propionate hydrogen bond would be expected to result in the destabilization of the heme pocket. The works of Choinowski et al. (1999) similarly revealed that the bond between the heme iron and the N^ɛ2^ atom of the proximal histidine residue in LiP is longer than that in CcP. The weaker iron‐nitrogen bond in LiP makes the heme more electron deficient thereby destabilizing the high oxidation states which has been suggested as a reasonable explanation for the higher redox potential of lignin peroxidase when compared to cytochrome c peroxidase.

## 6 Lignin Peroxidase Catalytic Reactions

LiP oxidizes different non‐phenolic lignin model compounds including β‐O‐4 linkage‐type arylglycerol‐aryl ethers. LiP oxidative properties involve the formation of radical cation through one electron oxidation and this action leads to side‐chain cleavage, demethylation, intramolecular addition, and rearrangements (Kirk, Tien, & Kersten, 1986; Miki, Renganathan, & Gold, 1986; Wong, 2009). Hydroxylation of benzylic methylene groups, oxidation of benzyl alcohols to their corresponding aldehydes or ketones, and phenol oxidation are other mechanistic oxidative process associated with LiP (Furukawa et al., 2014; Paliwal et al., 2012).

Besides the oxidation of non‐phenolic substrates, LiP possesses the additional capacity to oxidize a variety of phenolic compounds such as ring‐ and *N*‐substituted anilines (Baciocchi et al., 2001). Guaiacol, acetosyringone, catechol, vanillyl alcohol, and syringic acid are other phenolics susceptible to the oxidative potentials of LiP (Harvey & Palmer, 1990; Wong, 2009). At this juncture, it would suffice to state that veratryl alcohol, a non‐phenolic metabolite and high redox potential substrate has been suggested as a redox mediator (Christian et al., 2005) as it has been reported to enhance lignin peroxidase activity in lignin degradation (Lundell et al., 1993; Schoemaker, Lundell, Hatakka, & Piontek, 1994). The ability of LiP to oxidize lignin and other high redox potential compounds has been attributed to its exposed tryptophan residue (Trp^171^) which forms a tryptophanyl radical on the surface of the enzyme through long‐range electron transfer (LRET) to the heme. Also, variation in the tryptophan environment has been identified as a factor capable of modulating the enzyme activity, stability, and substrate specificity (Ivancich, Mazza, & Desbois, 2001). This, perhaps, accounts for the variation in the catalysis of VP and LiP as LiP is able to oxidize veratryl alcohol more effectively than VP. The ability of LiP is attributed to the acidic environment of Trp^171^ in *P. chrysosporium* LiP as it facilitates the stabilization of veratryl alcohol cation radical (Khindaria, Yamazaki, & Aust, 1996).

**Figure 2:**
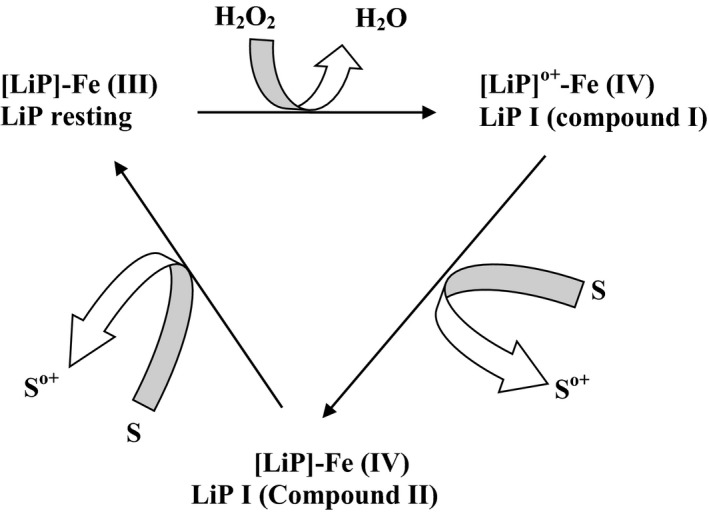
Catalytic reaction of lignin peroxidase. Adapted from Abdel‐Hamid et al. (2013)

The catalytic cycle of lignin peroxidase involves three steps (Fig. 2). The first reaction step is the oxidation of the resting ferric enzyme [Fe (III)] by hydrogen peroxide (H_2_O_2_) as an electron acceptor resulting in the formation of compound I oxo‐ferryl intermediate. In the second step, the oxo‐ferryl intermediate (deficient of 2e^−^) is reduced by a molecule of substrate such as non‐phenolic aromatic substrate (S) which donates one electron (1e^−^) to compound I to form the second intermediate, compound II (deficient of 1e^−^) while the last step involves the subsequent donation of a second electron to compound II by the reduced substrate thereby returning LiP to the resting ferric oxidation state which indicates the completion of the oxidation cycle (Abdel‐Hamid et al., 2013).

## 7 Contemporary and Prospective Functionalities of Lignin Peroxidase

Generally, peroxidases have been applied in soil detoxification (Mougin et al., 1994), treatment of phenols and chlorophenols polluted wastewaters (Cheng, Yu, & Zuo, 2006; Duarte‐Vazquez, Ortega‐Tovar, Garcia‐Almendarez, & Regalado, 2003), biopulping and biobleaching (Hatakka, Lundell, Hofrichter, & Maijala, 2003), development of biosensors to determine the presence of hydrogen peroxide and other related compounds (Hamid & Khalil‐ur‐Rehman, 2009; Jia et al., 2002) and in the development of skin‐lightening cream (Draelos, 2015). Most of these applications are yet to be commercialized. Worthy of note is the fact that biobulping, which is regarded as an effective alternative to chemical and mechanical pulping, is one of the oldest applications of peroxidases (Koshy & Nambisan, 2011). Catabolizing lignin in processed wood for paper production is the major role of LiP and other lignin modifying enzymes in the pulp and paper industry (Jaspers, Jimenez, & Penninkx, 1994; Michael, Dass, Grulke, & Reddy, 1991).

Recently, the applications of LiP have extended to the development of cosmeceutical and dermatological products. Most notable of these products are Melanozyme^™^ (lignin peroxidase based product) which is marketed as “*elure* ^*™*^ *skin brightening cream*” (www.elureskin.com) and Luminase, which serves as a catalytic skin tone illuminator, both manufactured by Syneron Medical Ltd, Irvine, California, USA. for the treatment of hyperpigmentation (sun spots or age spots) and skin lightening. The LiP used in the development of these skin‐lightening products has solely been derived from *P. chrysosporium*.

The potential applications of peroxidases in various other sectors have been envisaged (Hamid & Khalil‐ur‐Rehman, 2009), and interests in further exploit of these enzymes for industrial applications are on the increase. The, supposedly, high redox potential which bestows the desired functionality has been the reason for the endeared interest (Maciel, Silva, & Ribeiro, 2010). In the light of this knowledge, it becomes obvious that the prospective applications of LiP span through vast sectors of human endeavor including the biorefinery, textile, bioremediation, cosmetology, and dermatology. An overview of some functionalities is presented in succeeding sections.

## 8 Delignification of Feedstock for Ethanol Production

Ethanol is a good alternative to fossil fuel and as such, the use of lignocellulosic biomass as cheap source of feedstock for production of ethanol has continued to receive attention globally due to, in part, their renewable and eco‐friendly nature. Delignification of lignocellulose is an imperative step in the bioconversion of lignocellulose to ethanol and this process remains a challenge in lignocellulose biomass valorization. Biological method of delignification has been suggested as promising due to its mild reaction conditions, higher product yield, and low energy demand (Sánchez, Sierra, & Alméciga‐Díaz, 2011). But on the downside, it is saddled with long incubation period (in the order of several weeks to months) before reaching the same product (cellulose) recovery as it is obtained with the physical and chemical pretreatment methods (Khuong et al., 2014). Additionally, the utilization of carbohydrate as carbon source by the delignifying microbes has been shown to impact adversely on the quantity of recovered products (Sun, Li, Yuan, Yan, & Liu, 2011).

In a bid to overcome some of the challenges associated with microbial mediated bioconversion of lignocellulose, novel “lignocellulolytic enzyme system” has been suggested as an effective treatment strategy (Mukhopadhyay, Kuila, Tuli, & Banerjee, 2011; Wang et al., 2013). The suggested lignocellulolytic enzyme system includes LiP, MnP, and laccase among others and the associated merits of reaction specificity and, high product yield occasioned by the nonutilization of products as source of energy (Ma & Ruan, 2015; Wang et al., 2013). This makes the system a very promising model for industrial application. To buttress this position, Asgher, Ahmad, and Iqbal (2013) showed that ligninolytic enzymes (LiP, MnP, and Laccase) isolated from *P. ostreatus* IBL‐02 exhibited appreciable performance in sugarcane bagasse delignification as compared to sodium hydroxide (NaOH). However, the delignification functions attributed to the ligninolytic enzymes system (LiP, MnP, and laccases) from *P. ostreatus*, by Asgher et al. (2013), may not be associated to LiP as this fungus does not possess *lip* genes in its genome (Ruiz‐Dueñas, Fernandez, Martinez, & Martinez, 2011). However, if the position is to hold true, then, *lip* coding genes associated with the ligninolytic enzymes system shown by *P. ostreatus* would have been plasmid born. Otherwise, the delignification effect can be only attributed to laccases, MnP, and VP with MnP/LiP hybrid catalytic properties (Fernandez‐Fueyo et al., 2014).

## 9 Textile Effluent Treatment and Dye Decolourization

The textile industry consumes synthetic dyes significantly (Singh, Singh, & Singh, 2015), and these dyes are major sources of environmental pollution. Synthetic dyes such as azo, diazo, acidic, basic, reactive, disperse, metal‐complex, and anthraquinone‐based dyes are divers in structural variability (Christian et al., 2005). Understandably so, estimates of about 10–15% of dyes are lost in water during the process of textile dyeing (Asad, Amoozegar, Pourbabaee, Sarbolouki, & Dastgheib, 2007; Yanto, Tachibana, & Itoh, 2014). Subsequent release as effluent into various environment has also been estimated to amount to about 2–20%, thereby, portending a huge threat to public health (Yanto et al., 2014). To further bolster the danger posed by these textile dyes in the environment, many of these dyes and their degradation products have been declared toxic (Singh et al., 2015; Xu, Heinze, Chen, Cerniglia, & Chen, 2007). Hence, their presence in the environment should be a major concern. Therefore, effective and efficient removal strategy in the environment should be imperative. Consequently, various methods for dye decolourization and treatment of textile effluents have been developed. Some of these methods include adsorption, chemical treatment, ion‐pair extraction, coagulation, and flocculation techniques (Singh et al., 2015). These methods are not only effective but also costly and, they generate a great amount of sludge which may eventually create secondary pollution problem (Parshetti, Parshetti, Kalyani, Doong, & Govindwar, 2012). On the converse, biological method for dye treatment and removal including the use of microbes and macromolecular structures (enzymes) has been effective and it is saddled with less limitation. Studies on the applications of fungi and bacteria in dye abasement abound (Kumar & Sumangala, 2011; Shah, Patel, Nair, & Darji, 2013; Singh & Pakshirajan, 2010; Singh, Singh, & Singh, 2014), however, little attention has been given to the oxidative extracellular enzymes as an independent acting entity, thus, against this backdrop Ollikka et al. (1993) investigated the ability of some lignin peroxidase isozymes, isolated from *P. chrysosporium*, to decolourize azo, triphenyl methane, heterocyclic, and polymeric dyes in comparison with crude enzyme. The capability of the isolated isozymes of lignin peroxidase [LiP 4.65 (H2), LiP 4.15 (H7) and LiP 3.85 (H8)] to decolourize the test dyes in the presence of veratryl alcohol as a mediator was comparable to that of the crude enzyme which exhibited over 75% decolourization rate on the dyes. In another study by Abadulla, Robra, Gübitz, Silva, and Cavaco‐Paul (2000), the ability of enzyme preparations from some fungi (*Pleurotus ostreatus*; *Schizophyllum commune*; *Sclerotium rolfsii*; *Neurospora crassa*; *Polyporus* sp; *Trametes villosa*; and *Myceliophtora thermophile*) to decolourize a range of structurally different dyes was evaluated. It was discovered that the enzyme preparations effectively decolourize azo, triarylmethane, anthraquinone, and indigo dyes. Interestingly, the presence of lignin peroxidase increased the rate of decolourization by laccase in the study. Furthermore, Ferreira‐Leitao, Andrade de Carvalho, and Bon Elba (2007) compared the efficiency of fungal lignin peroxidase and plant horseradish peroxidase (HRP) for decolourization of methylene blue and its demethylated derivatives. It was shown that both enzymes were able to oxidize methylene blue and its derivatives. However, lignin peroxidase was reported to be more effective as its oxidation potential was almost double that of HRP. The authors (Ferreira‐Leitao et al., 2007) suggested that lignin peroxidase would be more suitable for degradation of phenothyazine dyes and decolourization of wastewater. Furthermore, a lignin peroxidase produced from sewage sludge treatment plant was reported to exhibit potential for textile effluent treatment and dye decolourization (Alam, Mansor, & Jalal, 2009; Singh et al., 2015). This was corroborated by Singh and Pakshirajan (2010) who attributed the high potential of *P. chrysosporium* in decolourization of colored wastewaters to efficient peroxidase enzyme system. In a recent study by Parshetti et al. (2012), purified lignin peroxidase from *Kocuria rosea* MTCC 1532 decolourized 11 different dyes belonging to various structural groups: azo, triphenyl‐methane, heterocyclic, polymeric, and metal‐complexes. Moreover, detoxification and decolourization of industrial waste by oxidative enzymes from bacteria and fungi have been reported (Rajasundari & Murugesan, 2011). The enzymes oxidize phenolic compounds to aryl‐oxy radicals creating insoluble complexes (Abdel‐Hamid et al., 2013). Other mechanisms of action of these enzymes include polymerization of contaminants and/or copolymerization with other nontoxic substrates to promote easy removal of the contaminants by other purification methods such as sedimentation, filtration, and adsorption (Gianfreda, Iamarino, Scelza, & Rao, 2006). This further indicates the potential of lignin peroxidase and other oxidative enzymes in textile and other industrial effluent treatment.

## 10 Coal Depolymerization and Degradation of other Xenobiotics

The ligninolytic enzyme system of microbes has been implicated in the degradation of several xenobiotics including chlorophenols, polycyclic aromatic hydrocarbons (PAHs), organophosphorus, and phenols (Marco‐Urrea & Reddy, 2012; Tisma, Zelic, & Vasic‐Racki, 2010; Wesenberg et al., 2003), and these compounds which are released from different anthropogenic sources are categorized as major environmental pollutants. Some of these compounds are active components of pesticides, disinfectants, herbicides, explosives, and dyes among others which are found in daily industrial application. Consequently, accumulation in the soil, ground water, and air constantly contaminates the environment and portend nuisance to public health (Wesenberg et al., 2003). Therefore, effective removal of these environmental pollutants is of utmost importance to stakeholders as well as the imperativeness for environmental health. Worthy of note is that extracellular peroxidases, from ligninolytic microbes, have been reported to play a significant role in the degradation of xenobiotic compounds (Duran, Deschler, Precigou, & Goulas, 2002). LiP, as part of the ligninolytic enzyme system of both fungi and bacteria, has been reported to mineralize different types of recalcitrant aromatic compounds including three‐ and four‐ring polycyclic aromatic hydrocarbons (Gunther, Sack, Hofrichter, & Latz, 1998; Wesenberg et al., 2003), polychlorinated biphenyl (Krcmar & Ulrich, 1998; Wesenberg et al., 2003), chlorophenols (Antonopoulos, Rob, Ball, & Wilson, 2001), and synthetic dyes (Chivukula, Spadaro, & Renganathan, 1995; Wesenberg et al., 2003). These articulated data show the suitability of lignin peroxidase in bioremediation.

Furthermore, studies have shown that unburnt coal can have negative effect on water quality and the functioning of the aquatic ecosystem (Ahrens & Morrisey, 2005). Countries in the categorization of large coal producing, consuming, and/or exporting are confronted with the challenge of managing the impact of coal in the environment. The presence of coal in water has been suggested as a source of increased salinity, acidity, trace metals, hydrocarbons, and chemical oxygen demand (Milani, Reynoldson, Cheam, Garbai, & Rajkumar, 1999; Stephan, 2010; Ward, 2002). Toxic polycyclic aromatic hydrocarbons (PAHs) from unburnt coal have also been suggested as an important source of contamination in the aquatic environment (Achten & Hofmann, 2009). PAHs from the incomplete combustion of coal have been implicated in the pollution of abandoned coal gasification site (Surtherland, Raffi, Khan, & Cerniglia, 1995) and soil pollution. They are also found at high concentration in the bottom of sediments of water bodies. The presence of coal particles in soils and sediments can result from coal mining and transportation (Achten, Cheng, Straub, & Hofmann, 2011; Johnson & Bustin, 2006). Given the increased coal mining operations in coal‐producing and consuming countries, toxic PAHs as contaminants in water, sediments, and soil are continually emerging. Consequently, investigation into the potentials of lignin peroxidase and other ligninolytic enzymes in the degradation of polycyclic aromatic hydrocarbons, known and emerging in the environment should be explored. Perhaps, of great interest shall be the depolymerization of PAHs emanating from coal industrial applications and utilizations.

## 11 Melanin Oxidation—Novel Cosmetic Lightening Agents

Melanin, produced by melanocytes in a process termed melanogenesis and stored in melanosomes, is the dark pigment responsible for human skin and hair colouration. The melanosomes are transferred to keratinocytes (epidermal cells) for onward transportation to the upper layer of the epidermis to confer on the skin its typical color (Mauricio, Karmon, & Khaiat, 2011). Its deficiency can lead to various diseases and disorders including albinism which is the absence of melanin pigment in an individual. Pigmentation disorders such as hyperpigmentation, a common dermatologic condition (Kindred, Okereke, & Callender, 2013) that affects all skin types, have been attributed to the accumulation of melanin in the upper layer of the epidermis (Mauricio et al., 2011; Simon, Peles, Wakamatsu, & Ito, 2009). Basically, melanin is divided into two types: eumelanin (brown and black) and pheomelanin (red or yellow). Eumelanin is reported to be the more ubiquitous melanin type in mammals, as it is found in different parts of the body such as hair, skin, inner ear, eye, and brain (Khammuang & Sarnthima, 2013).

The impact and distribution of melanin is not limited to mammals, it is also found in many other life forms including plants and microbes where they serve different functions. Some of the reported biological functions of melanin include protection against environmental stress (Kogej et al., 2007; Liu & Nizet, 2009), increased antibiotic resistance in bacteria (Lin et al., 2005), and involvement in fungal pathogenesis of plants (Butler, Gardiner, & Day, 2005; Khammuang & Sarnthima, 2013). Melanin is known to be very durable and its durability has been attributable to its complex structure. Its basic structural unit is represented by covalently linked indoles. In addition, melanin is a heterogeneous polymer composed majorly of dihydroxyindole units which exist as a mixture of both catechol and quinone (Prota, 1992; Woo, Cho, Lee, & Kim, 2004). The structural characteristics of melanin is comparable to that of lignin and coal wherein the polymers are made up of indole and phenolic subunits (Woo et al., 2004), hence its resistance to degradation.

**Figure 3:**
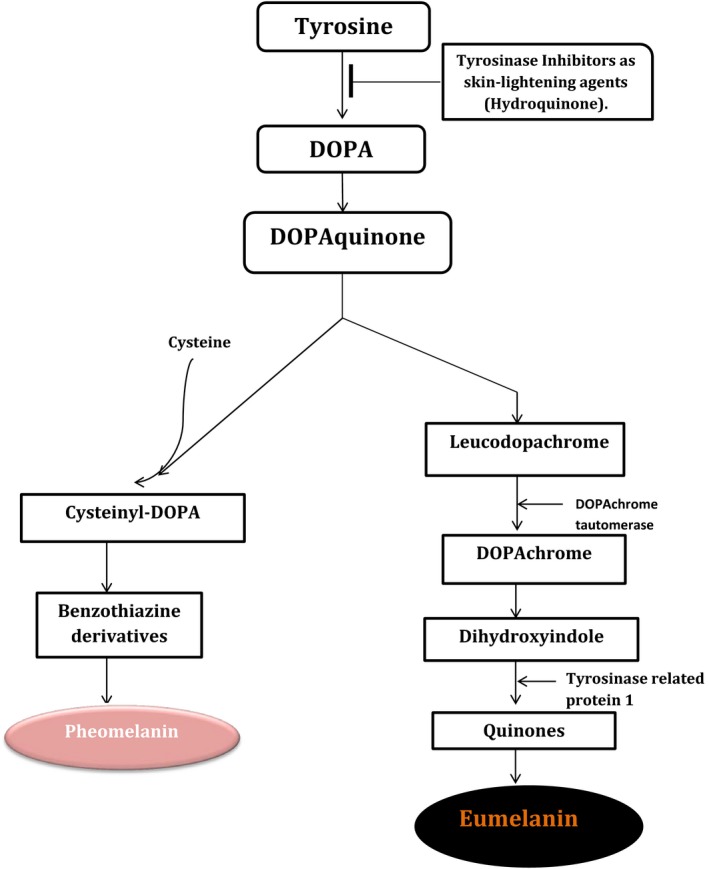
Pathway of melanin biosynthesis

Although one of the biological functions of melanin in human may be to protect the underlying tissues from harmful ultraviolet (UV) radiation (Krol & Liebler, 1998), many hyperpigmented women in Africa and other black nations desire a light face and skin as the Caucasians desire a spotless skin. To achieve this desire, cosmeceutical and dermatological industries have developed treatments for skin lightening employing the following mechanisms of action: prevention of melanogenesis by inhibiting tyrosinase, an enzyme that catalyzes the rate‐limiting step [conversion of tyrosine to dihydroxyphenylalanine (DOPA)] in melanin biosynthesis (Kim & Uyama, 2005) as illustrated in Fig. 3, preventing the stimulation of melanocytes by ultraviolet A radiation, activation of cell turn‐over (Woo et al., 2004), and blocking the transfer of melanosomes to keratinocytes (Mauricio et al., 2011).

Hydroquinone, described as the gold standard in the treatment of hyperpigmentation (Kindred et al., 2013) has been the most effective skin‐lightening agent. However, its safety has generated a lot of controversy and concern. This has motivated research into exploration of alternative agents for the treatment of skin pigmentation disorders including melasma. Currently, some of the available alternative skin‐lightening agents include mequinol, topical retinoids, azelaic acid, arbutin and deoxyarbutin, kojic acid, licorice extract, ascorbic acid, soy, aleosin, niacinamide, and *N*‐acetylglucosamine (Kindred et al., 2013). Hydroquinone and most of these alternatives operate through tyrosinase inhibition mechanism (Grimes, 2009), probably by binding directly to the enzyme or interacting with the copper molecules at its active site (Sheth & Pandya, 2011) thereby reducing the conversion of DOPA to melanin. However, skin‐lightening by inhibition of melanin synthesis is slow in achieving the desired results (Woo et al., 2004). Hence, there is the need to explore alternative agents with the potential to directly decolourize melanin pigment through oxidation as a means of skin‐lightening potential. Perhaps, the ability of ligninolytic enzymes to oxidize a wide range of structurally different substrates makes them suitable candidates for the oxidation of melanin which is structurally similar to lignin. Thus, ligninolytic enzymes with melanolytic ability have the potential for application in the cosmetics industry.

Woo et al. (2004) demonstrated that crude lignin peroxidase from *P. chrysosporium* could decolourize synthetic melanin, thus suggesting its application in the development of new cosmetic lightening agents. Furthermore, Mohorčič et al. (2007) produced melanolytic enzyme capable of degrading human skin melanin from *Sporotrichum pruinosum* and peroxidases from *Ceriporiopsis* sp. Strain MD‐1 have also been reported to decolourize synthetic and human hair melanins (Nagasaki et al., 2008). Similarly, the study reported by Khammuang and Sarnthima (2013) shows that crude laccases from *Lentinus polychrous* Lév. was able to decolourize synthetic melanins. The enzyme was reported to be more effective in the presence of ABTS [2‐2'‐azino‐bis (3‐ethylbenzthiazoline‐6‐sulphonic acid)] as a mediator. Perhaps, it would be noteworthy to state that all the previously studied melanolytic enzymes are of fungal origin, thus, an exploration into bacterial melanolytic enzymes for application in the development of skin care products shall be a novel concept.

**Figure 4:**
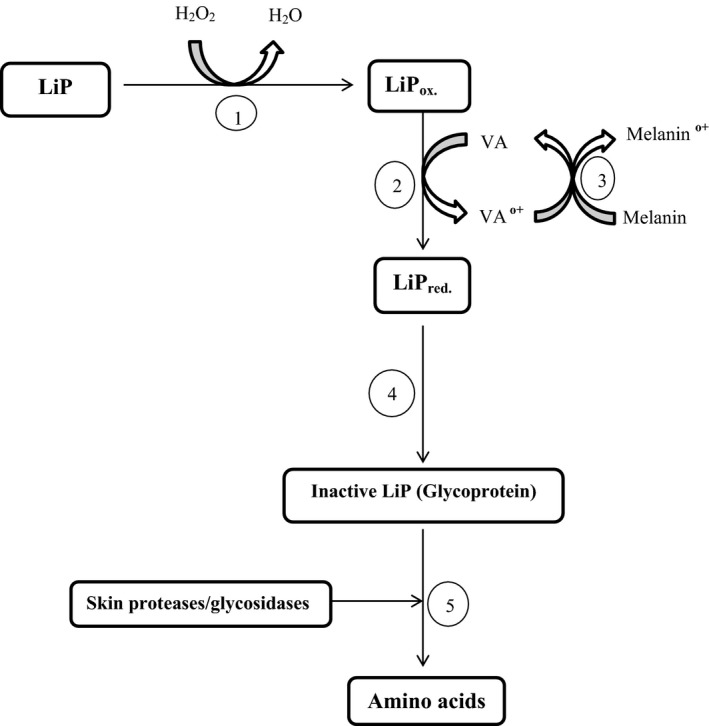
Mechanism of action of lignin peroxidase as cosmetic lightening agent. Step 1; oxidation of LiP by hydrogen peroxide, Step 2; reduction of oxidized LiP by one molecule of veratryl alcohol (VA), Step 3; oxidation of melanin, Step 4; inactivation of LiP by change in pH to become a simple glycoprotein, Step 5; hydrolysis of glycoprotein into amino acids by proteases and other glycosidases naturally present in the skin

As the proposition for the use of lignin peroxidase as an alternative to hydroquinone cream increases, efforts are being made to ascertain efficacy and safety of these compounds both at an acute and chronic phase. Consequently, Mauricio et al. (2011) evaluated the skin‐lightening efficacy and safety of lignin peroxidase (LiP) constituted cream in comparison with 2% hydroquinone cream in Asian women. It was observed in the study that the application of LiP cream provided a significantly faster and observable skin‐lightening effect than 2% hydroquinone cream which led to the overall preference of LiP creams. LiP has demonstrated a skin‐lightening effect comparable to that of hydroquinone, with no observable adverse effect, and with superiority in skin texture and roughness (Draelos, 2015). However, more studies are required to compare LiP‐based cream with higher concentrations of hydroquinone and its efficiency in the treatment of other pigmentary disorders. The mechanism of action of LiP as cosmetic lightening agent involves five steps (Fig. 4). The first reaction step is the oxidation of LiP (the active component of cosmetic lightening cream) by hydrogen peroxide (an activator, which activates the enzyme on application on the skin) as in a typical catalytic reaction of LiP. Step 2 involves the reduction of oxidized LiP by a molecule of veratryl alcohol (VA), a substrate specific for LiP, leading to the production of a veratryl alcohol radical (VA^**o+**^) which in turn mediates the oxidation of melanin on the skin in step 3. In step 4, LiP is inactivated by change in pH which occurs as a result of application of the enzyme on the skin, thereby becoming a simple glycoprotein which is subsequently hydrolyzed into amino acids by proteases and other glycosidases naturally present in the skin in the last step (step 5).

## 12 Prospects in Drug Discovery

Melanin has been reported to protect microbes against environmental stress (Kogej et al., 2007; Liu & Nizet, 2009), and also increase antibiotic resistance in bacteria (Lin et al., 2005). To buttress this point, the activities of the some antibiotics (kanamycin, ampicillin, polymyxin B, and tetracycline) against *Escherichia coli* were shown to be significantly decreased in the presence of melanin (Lin et al., 2005). The decreased antibacterial activity was attributed to the interaction of the antibiotics with melanin, but the mechanism of the antibiotics–melanin interaction is yet to be understood. The understanding of the mechanism of this interaction is therefore suggested as this could give an insight into drug interaction and further proffer solutions on how to improve the efficacy of antibiotics in the presence of melanin. More so, an intensive study of bacterial melanogenesis seems to be a promising research area toward drug discovery against antibiotic‐resistant pathogenic bacteria which has melanin as a virulence factor (Kurian & Bhat, 2014; Plonka & Grabacka, 2006).

## 13 Conclusion

The prospects of LiP in biorefinery, bioremediation, cosmetology, and dermatology among other endeavor of human activities cannot be overemphasized, and its potential as a suitable alternative to hydroquinone in the development of skin‐lightening cream and treatment of hyperpigmentation has been properly synopsized in this review. Besides, the prospects of other ligninolytic enzymes systems, not yet known, abound in all the aforementioned industries and beyond. In view of the articulated importance and prospective applications of LiP, the exploration of the underexplored microbial diversity for novel LiP with enhanced capabilities in solving problems as envisaged in this review becomes pertinent. Besides, the need of LiP for remediation of toxic phenolics among others in the environment and its application in cosmetics and new drugs discovery, jobs, and economy will be boosted, thus improving upon the social standing of any community with significant inroad into this lucrative bioeconomic sector.

## Funding Information

We gratefully acknowledge the financial support from the National Research Foundation (NRF) under the South Africa/Tunisia bilateral collaboration (grant number: 95364), the Govan Mbeki Research and Development Centre (GMRDC) of the University of Fort Hare and South Africa Medical Research Council (SA‐MRC).

## Conflict of Interest

Authors declare that there are no conflicts of interest.
